# Inositol Analysis by HPLC and Its Stability in Scavenged Sample Conditions

**DOI:** 10.4172/2161-0444.1000246

**Published:** 2015-02-28

**Authors:** Robert M Ward, John Sweeley, Ralph A Lugo

**Affiliations:** 1 Divisions of Neonatology and Pediatric Pharmacology, Adjunct Professor, Pharmacology/Toxicology, University of Utah, 295 Chipeta Way, Salt Lake City, UT 84108, USA; 2 Department of Pediatrics, University of Utah, 417 Wakara Way, Salt Lake City, UT 84108, USA; 3 Department of Pharmacy Practice, Gatton College of Pharmacy, East Tennessee State University, Johnson City, Tennessee 37614, USA

## Abstract

Inositol is a 6-carbon sugar alcohol that has been shown in limited studies to reduce retinopathy of prematurity and chronic lung disease in premature newborns. Developmentally it has a high concentration in the fetus that decreases with gestational age. It is transported from the fetus to the mother across the placenta. Although studies are underway to determine inositol kinetics in premature newborns treated therapeutically, the effects of gestational age, age after birth, and feeding on inositol concentrations after birth have not been studied adequately in premature newborns. Such studies would minimize blood removal and trauma in preterm newborns by using plasma samples scavenged from the clinical laboratory to measure inositol after birth, if they remain stable. This report describes a new high pressure liquid chromatographic assay for inositol and its use to study the stability of inositol in conditions of storage that might be encountered within the clinical laboratory. The assay is linear from 0 to 1000 Mm with a lower limit of quantitation of 50 μM. Inositol in human plasma remains stable in refrigeration and at room temperature for up to 14 days and is not affected by storage in red blood cells that are intact or lysed. Anticoagulants encountered in clinical blood samples do not interfere with the chromatograms. Thus, it is feasible to measure the changes in inositol concentrations in plasma from preterm newborns that is scavenged from the clinical laboratory after storage for as long as 14 days.

## Background

In previous studies among preterm infants with RDS, intravenous myo-inositol (referred herein as inositol) reduced death, broncho pulmonary dysplasia and retinopathy of prematurity [1-3]. Inositol is endogenously synthesized from glucose, and catabolized by inositol oxidase in the renal cortex, an enzyme with low activity at birth [4,5]. Inositol is a6-carbon, non-reducingsugar alcohol thatis a component of surfactant and also participates widely in intracellular signaling as phospho-inositides [6,7]. It has the same molecular formula and thus molecular weight as glucose. The most commonly occurring form is *cis*-1,2,3,5-*trans*-4,6-cyclohexanehexol, known as myo-inositol. The function of inositol in utero is not clear. Fetal levels are high early in pregnancy and fall with increasing gestation; inositol is transported to the mother across the placenta [8,9]. The pharmacokinetics of inositol during treatment of preterm newborns is being investigated, but the physiologic changes in inositol after preterm birth and their relation to intake through feeding or intravenous supplementation have not been described in detail. Study of the pharmacokinetics of inositol in extremely premature newborns with limited blood volume is best carried out using unused plasma samples scavenged from the clinical laboratory after analysis of clinically indicated studies. Such studies require that the stability of inositol in plasma in the conditions used in storage within the laboratory be known. However, to date, there are no published studies evaluating the stability of inositolin these conditions.

## Objective

The objective of this study was to develop an assay for inositol using high pressure liquid chromatography and describe the 14-day stability of inositol in plasma stored at room temperature, under refrigeration at 4°C, and frozen at -80°C,as well as the effects of hemolysis and various anticoagulants.

## Methods

An analytic technique for myo-inositol was developed and validated using high-pressure liquid chromatography (HPLC). Using this analysis, the stability of plasma inositol was measured for up to 14 days in samples stored at room temperature, refrigerated at 4°C, and frozen at -80°C in a range of concentrations expected to occur clinically, 100 to 700 μM. The effects of different anticoagulants and storage with intact and lysed red blood cells were also tested.

### HPLC analysis

**Figure 1:**
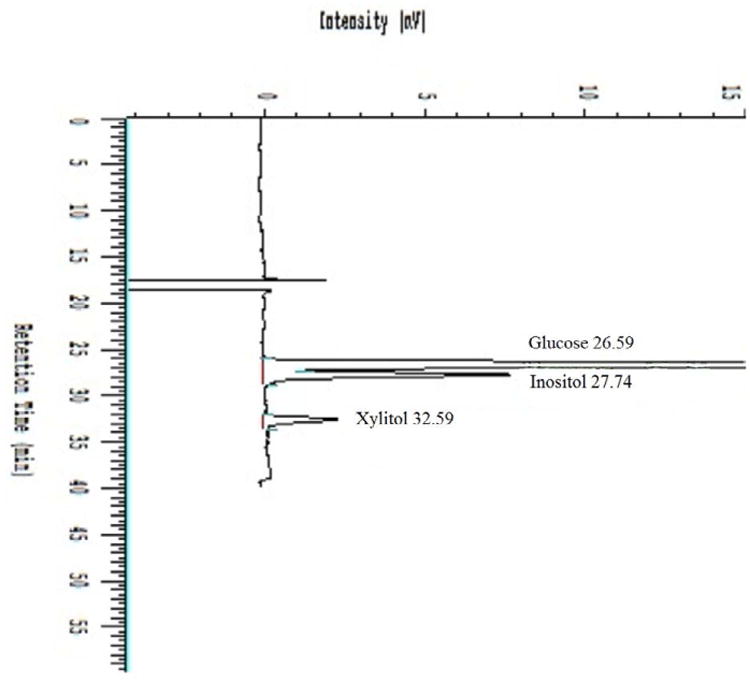
Typical chromatogram using the conditions described in the manuscript, showing inositol eluting at 27.74 minutes, xylitol eluting at 32.59 minutes and glucose eluting at 26.59 minutes.

The HPLC system consisted of an Hitachi L-6200A pump controlled by Hitachi D-6000 Chromatography Data Station Software, which collected and analyzed the chromatography data. Detection was achieved with a Waters 410 Differential Refractometer. Separation was achieved with two Bio-Rad Aminex HPX-87H (300×7.8mm) columns connected in tandem and maintained at 65°C in a separate column heater. All chemicals were HPLC grade and purchased from Sigma-Aldrich Chemical Company (St. Louis, MO). The mobile phase was 0.02 M H_2_SO_4_ at a flow rate of 0.4 ml/min. Retention times under these conditions were 27.4 min for inositol, 31.4 min for xylitol (internal standard) and 26.9 min for glucose the largest, potentially interfering peak (Figure 1). Plasma standards and samples were prepared for HPLC analysis by transferring 100 μL of sample or standard to a 1.5 ml Eppendorf tube, to which was added 500 μLof an aqueous solution of xylitol at 400 μM. This mixture was mixedbriefly before adding 100 μL of 0.3 N Ba(OH)_2_ followed by 100 μL of 0.3 N Zn(SO)_4_. The tube was capped, vortexed vigorously for 20 seconds and centrifuged @ 8000 g for 8 minutes. The clear supernatant was transferred to an autosampler vial from which 50 μL was injected into the HPLC system for analysis. The standard curves were developed from inositol concentrations of 0, 200, 400, 600, and 1000 μM prepared in human plasma obtained from the Associated Regional University Pathologists blood bank (Salt Lake City, Utah), divided into 1 mL aliquots and stored frozen at –70°C for 2 days or less. The linearity was determined by linear regression of inositol concentration on the basis of peak area ratios of inositol to the internal standard.

### Assay accuracy and precision

To determine the accuracy and precision of the assay, samples of human plasma were spiked with inositol in concentrations of 50, 100, 300 and 700 μM and frozen in 1 ml aliquots at -80°C. Accuracy, defined as the difference from the expected concentration measured by percent error[(analyte concentration – expected concentration)/expected concentration × 100], was determined for these 4 concentrations analyzed on 6 different days. Precision, defined as the coefficient of variation (CV, which was calculated by standard deviation/mean × 100) among analyses of the same concentration, was evaluated by repeated analysis of samples from inositol concentrations of 100 μM, 300 μM and 700 μM in 6 batches on a single day. All analyses were carried out with duplicate injections and averaged. New standard curves were determined on each day using a 1 mL aliquot of each standard that was stored frozen at -80°C.

### Inositol stability

The effects of temperature and time on inositol stability in plasma were tested at room temperature, under refrigeration at 4°C and frozen at -80°C for 0 to 14 days. Using human plasma (containing heparin 10 units/ml), inositol was added to yield a final concentration of 500 μM. The plasma was divided into three sets of fifteen 500 μLsamples. One set was stored at -80°C, one set was refrigerated at 4°C and another was stored at room temperature approximately 21°C. Five samples were analyzed from the room temperature and refrigerated sets and compared to the concentration from the samples frozen at -80°C on day 0, day 7 and day 14. New standard curves were prepared in human plasma on each day of the analysis.

### Effects of whole and lysed blood on inositol concentrations

Heparinized (10 U/ml) whole blood samples were prepared by adding inositol to yield a final concentration of 1000 μ Min fifteen 500 μL replicates. The first set of 5 replicates was immediately centrifuged, separated, and the plasma was frozen at -80°C. The second and third sets of 5 samples were allowed to remain at room temperature for 1 hour and 24 hours, respectively. They were then sonicated for 20 minutes to lyse the cells and the plasma was frozen at -80°C. Samples were analyzed using standards prepared as abovein human plasma.

### Effects of anticoagulants

Blood was collected into tubes containing potassium ethylenediaminetetra acetic acid (EDTA), sodium heparin, or lithium heparin after which it was centrifuged and the plasma removed and prepared for analysis. The chromatograms from samples prepared from each type of anticoagulant were compared to that from xylitol and inositol to detect whether there were interfering peaks or similar retention times.

## Results

### HPLC analysis

The Figure shows a typical chromatogram with the large glucose peak eluting immediately prior to inositol. Although the chromatogram did not always reach baseline between the glucose and inositol peaks, the standard curves were always linear using the ratios of peak area of inositol/xylitol. Themean correlation coefficient for the inositol standard curves was 0.997 ± 0.002(n=12). For inositol concentrations of 50, 100, 300, and 700 μM, the interdayprecision (coefficient of variation) on 6 different days was 12.65% for 50 μM,6.95% for 100 μM, 2.67% for 300 μM, and 1.90% for 700 μM (Table 1).Accuracy (% error) from analyses on 6 days averaged 16.28 ± 14.78% for 50 μM90.26 ± 6.97% for 100 μM, -1.59 ± 2.63% for 300 μM, and -0.72 ± 1.54% for 700 μM. The intraday variation in analyses was determined from 6 analyses of inositol concentrations of 100, 300 and 700 μM determined on the same day. These showed an intraday precision (coefficient of variation) of 0.72% for 100 μM, 0.14% for 300 μM, and 1.58% for 700 μM (Table 2). Accuracy (% error) averaged -10.7% ± 0.6% for 100 μM, -6.50 ± 0.1% for 300 μM, and -4.10 ± 1.50% for 700 μM.

Stability testing demonstrated that a storage temperature of 4°C and 21°C had no effect on the concentration for as long as 14 days. At room temperature, 5 analyses of 500 μM concentrations yielded a CV 3.3% on day 0 (507.3 ± 7.4 μM); CV 1.5% on day 7 (499.9 ± 8.0 μM); CV 1.6% on day 14 (499.9 ± 1.6 μM). Similarly, with refrigeration to 4°C, 5 analyses of 500 μM concentrations yielded a CV 2.0% on day 0 (502.6 ± 9.8 μM); CV 3.4% on day 7 (508.8 ± 17.2 μM); and CV 5.6% on day 14 (492.8 ± 27.6 μM). Storage of inositol in whole blood for up to 24 hours did not alter the plasma concentration. Five inositol samples of 1000 μM analyzed at three time points resulted in a CV 4.8% at time 0 (1088.8 ± 51.9 μM); CV 3.4 % at 1 hour (1062.9 ± 36.1 μM); and CV 6.0% at 24 hours (1034.4 ± 61.6 μM). Storage of inositol in lysed red blood cells also did not alter the concentration. In 5 samples of inositol 1000 μM, the CV was 2.5% at time 0 (1122.2 ± 28.6 μM); CV 7.8% at 1 hour (1091.6 ± 84.8 μM); and CV 11.5% at 24 hours (1032.1 ± 119.2 μM).

None of the anticoagulants, including EDTA, sodium heparin and lithium heparin interfered with the peaks for inositol or xylitol (data not shown).

## Discussion

Excess plasma stored in the hospital chemistry laboratory after completion of clinically indicated tests is designated a scavenged sample. The date and time of sample collection is labeled on the remaining plasma. With the limited total blood volume in extremely low birth weight newborns of 36 to 45 ml, the use of scavenged samples is particularly attractive. It also obviates additional trauma from blood sampling by heel stick or venipuncture in these patients who already undergo a large number of painful procedures.^10^ Although the use of scavenged blood samples is particularly applicable to studies in newborns, it has not been widely used. Wade et al. [11] used scavenged plasma samples to provide 39% of the concentration time points in a study of the population pharmacokinetics of fluconazole in preterm newborns. They demonstrated that the samples were stable for at least 72 hours in the storage conditions present in the clinical laboratory [12].

Not all drugs remain stable in plasma. The esterases in plasma are known to breakdown some drugs that are administered as prodrug esters, such asbeclomethasonedi propionate [13] and bendamustine [14]. Other drugs, such as ampicillin are known to be unstable at room temperature as well as during refrigeration [15].

Treatment of preterm newborns with inositol is underway to try to reduce chronic lung disease and retinopathy of prematurity based on recent pharmacokinetic studies [16]. Another study is planned to determine the variations and changes in inositol concentrations at birth and during the first days to weeks in extremely premature newborns using scavenged samples from the clinical laboratory. Breast milk contains inositol and some infant formulas are supplemented with inositol, as well. The influence of the amount and composition of feeding on plasma inositol concentrations will help determine the biologic variability of inositol in this population that is at risk for chronic lung disease and whether there is any correlation between these clinical problems and their inositol concentrations at birth.

The current study demonstrates that inositol can be accurately and precisely analyzed by HPLC in concentrations from 50 to 700μM even though high blood concentrations of glucose prevent complete return of the chromatographic curve to baseline. At 50 μM, precision and accuracy are reduced but still acceptable for analysis of the changes in inositol after birth. For concentrations less than 50 μM, inositol must be analyzed with other techniques, such as LC/MS/MS for accurate determination.

Scavenged samples stored in the clinical laboratory either in the refrigerator or at room temperature for up to 14 days retain inositol without degradation. Similarly, storage with red blood cells for 24 hrs without separation does not alter the concentration. When inositol was stored in lysed red blood cells, the concentration increased 11.5% at 24 hours which may fall outside the acceptable range of variation for some studies. None of the anticoagulants demonstrated peaks in the vicinity of those for xylitol or inositol.

## Conclusion

This analytic technique for inositol by HPLC can be used for analysis of human plasma samples retrieved from storage in the clinical laboratory up to 14 days after removal from the patient. Storage in red blood cells that are not lysed does not change the concentration, but storage in lysed red blood cells increases the concentration by 11.5% at 24 hours.

## Figures and Tables

**Table 1:** Interday Precision (Coefficient of Variation (CV, standard deviation/mean × 100)) and Accuracy (Percentage Error (%error) = (measured concentration – expected concentration)/expected concentration × 100)) for Inositol Analyses in Concentrations of 50 to 700 μM, analyzed on 6 different days, in columns.

		Measured Concentrations of Inositol (μM)								
Expected Concentration	Day:	1	2	3	4	5	6	Mean	SD*	CV*
700 μM		670.8	673.4	671.5	673.2	678.1	707.5	679.1	12.91	1.90
	% error:	-4.17	-0.04	-0.04	-0.04	-0.03	0.01	-0.72	1.54	
300 μM		295.5	291.0	287.3	296.9	289.5	311.2	295.2	7.89	2.67
	% error:	-1.5	-3.0	-4.2	-1.0	-3.5	3.7	-1.59	2.63	
100 μM		97.7	90.1	93.1	106.8	107.0	106.7	100.3	6.97	6.95
	% error:	-2.3	-9.9	-6.9	6.8	7.0	6.7	0.26	6.97	
50 μM		42.6	64.3	62.6	59.5	63.3	58.2	58.4	7.39	12.65
	% error:	-14.8	28.7	25.3	19.0	26.5	16.3	16.84	14.78	

* SD = standard deviation, CV = Coefficient of Variation

**Table 2:** Intraday Precision (Coefficient of Variation (CV) = standard deviation/mean × 100)) and Accuracy (Percentage Error (%error) = (measured concentration – expected concentration)/expected concentration × 100)) for 6 analyses of inositol on the same day in concentrations of 100 to 700 μM.

		Measured Concentrations of Inositol (μM)								
Expected Concentration	Analysis No.:	1	2	3	4	5	6	Mean	SD*	CV*
700 μM		673.6	669.6	667.1	659	667.8	690.7	671.3	10.6	1.58%
	%error:	-3.77	-4.34	-4.7	-5.85	-4.6	-1.33	-4.1	1.5	
300 μM		280.2	280.8	271.1	283.9	289.7	290.5	280.5	0.4	0.14%
	%error:	-6.59	-6.4	-9.65	-5.36	-3.44	-3.16	-6.5	0.1	
100 μM		89.8	88.9	85.8	88	87.9	93.2	89.3	0.6	0.72%
	%error:	-10.21	-11.12	-14.24	-11.98	-12.13	-6.8	-10.7	0.6	

* SD = standard deviation, CV = Coefficient of Variation
